# Sustainable Development of Water Quality, Growth, and Production Efficiency of the Giant Mottled Eel (*Anguilla marmorata*) at Different Stocking Densities in the Indoor Media-Based Aquaponics System

**DOI:** 10.3390/ani15182705

**Published:** 2025-09-15

**Authors:** Prosun Roy, Dae Hwan Lee, Sung Min Choi, Yun Keun An, Sang Duk Choi

**Affiliations:** 1 Department of Big Data Fishery Resource Management Interdisciplinary Program, College of Fisheries and Ocean Sciences, Chonnam National University, Yeosu 59626, Republic of Korea; prosunroy3001@jnu.ac.kr; 2 Department of Fisheries Science, College of Fisheries and Ocean Sciences, Chonnam National University, Yeosu 59626, Republic of Korea; henry7595@naver.com; 3 Department of Aquaculture, College of Fisheries and Ocean Sciences, Chonnam National University, Yeosu 59626, Republic of Korea; blackcanon2@naver.com (S.M.C.); yunkeunan@yahoo.co.kr (Y.K.A.); 4 Institute of Fishing Village & Aquaculture, Chonnam National University, Yeosu 59626, Republic of Korea

**Keywords:** *Anguilla marmorata*, density-dependent, indoor aquaponics, water parameters

## Abstract

The sustainability of aquaponic systems, particularly regarding *Anguilla marmorata* productivity and water quality, is strongly influenced by stocking density. This study investigated the effects of different stocking densities on water quality, growth performance, and the proximate composition of *A. marmorata* in a media-based indoor aquaponics system cultivated with *Brassica rapa* var. *pekinensis*. The 8-week trial tested stocking densities of 10 (T_1_), 15 (T_2_), and 20 (T_3_) eels 250 L^−1^, each with three replicates. Significant differences (*p* < 0.05) were observed in water parameters and nutrient accumulation, with higher densities linked to greater nutrient availability. Growth performance indicated that T_2_ achieved the best overall results, with significantly higher weight gain, specific growth rate, and feed efficiency and the lowest feed conversion ratio of *A. marmorata* and enhanced plant growth (*p* < 0.05). T_1_ showed the highest protein efficiency ratio, while T_3_ yielded the highest biomass per volume. Moisture and crude protein contents were highest in T_3_, whereas crude lipid and ash contents were highest in T_1_ and T_2_, respectively (*p* < 0.01). These findings suggest that a moderate stocking density (15 eels 250 L^−1^ of water) optimizes fish and plant growth, system sustainability, and nutrient utilization in small-scale aquaponic systems.

## Simple Summary

Aquaponics is a sustainable agricultural approach that integrates aquaculture (fish farming) with hydroponics (growing crops) and plays a vital role in sustainable food production. High stocking densities can result in resource competition, poor fish growth, and deteriorated water quality, while low densities may lead to resource underutilization and reduced productivity, negatively impacting both fish and plant growth. This study sought to evaluate how the number of *Anguilla marmorata* per tank (stocking density) affects eel growth and water quality in a media-based aquaponics system with *Brassica rapa* var. *pekinensis*. Our findings revealed significant variations in water parameters and nutrient levels among treatments, with higher stocking densities increasing nutrient availability. Notably, medium stocking density produced the most favorable growth performance metrics. Medium and high stocking densities also showed significant differences in proximate composition, with medium stocking density yielding the highest feed intake and protein efficiency and high stocking density producing the greatest biomass. These findings suggest that a moderate stocking density of *A. marmorata* is optimal for enhancing fish growth, system efficiency, and nutrient utilization while simultaneously supporting the growth of *B. rapa* var. *pekinensis* within indoor media-based aquaponics. This research contributes valuable insights toward developing more efficient and sustainable food production systems, especially in resource-limited environments.

## 1. Introduction

In response to the growing global demand for fish and plant products, aquaponics is gaining recognition as a sustainable and efficient solution to address food shortages and water scarcity. Aquaponics is an integrated agricultural system that combines recirculating aquaculture with hydroponics, enhancing productivity and water-use efficiency by increasing crop yields without additional water input. Simultaneously, it reduces dependence on chemical fertilizers and pesticides, offering both environmental and social benefits [1]. In aquaponic systems, effluents from the aquaculture unit serve as a nutrient source for hydroponically grown plants, effectively contributing to water purification and overall system sustainability [2]. This process is facilitated by a consortium of nitrifying autotrophic bacteria, primarily nitroso-bacteria (e.g., *Nitrosomonas* spp.) and nitro-bacteria (e.g., *Nitrospira* and *Nitrobacter* spp.) [3]. The nitroso-bacteria oxidize ammonia into nitrite, which is then converted into nitrate by the nitro-bacteria [4]. The resulting nitrate serves as a vital nutrient for plant growth [4].

The efficiency of an aquaponic system depends on harmonizing the needs of both fish and plants through careful management of environmental factors such as temperature, pH, and nutrient availability [5]. Among these, stocking density is a critical parameter that significantly influences water quality and, consequently, overall system performance [6]. High stocking densities can increase competition for space and feed, alter fish growth, behavior, metabolic activity, and immune responses, and degrade water quality due to the buildup of waste products [7,8]. Conversely, low stocking densities may lead to underutilized system resources and reduced productivity. An imbalanced stocking density also disrupts the nutrient-to-plant ratio, potentially hindering plant growth or compromising fish welfare, depending on the system’s design and capacity [9,10,11]. Therefore, identifying the optimal stocking density is essential to ensure biological and environmental compatibility while maximizing system efficiency.

Eels, particularly the giant mottled eel (*Anguilla marmorata*), have demonstrated strong adaptability to diverse environmental conditions and intensive aquaculture systems, making them a promising candidate for aquaponics [12]. *A. marmorata* is a tropical freshwater species known for its exceptional growth potential, reaching up to 2 m in length and weighing as much as 21 kg [13]. It is the most broadly distributed among all freshwater eel species and subspecies, extending from the eastern coast of Africa to the Marquesas Islands in the southeastern Pacific, and northward to southern Japan [14]. In Japan’s Yakushima Island, *A. marmorata* is commonly found inhabiting rocky freshwater streams and rivers, where it shelters in crevices between boulders and cobbles [15,16]. Due to its strong disease resistance and compatibility with intensive aquaculture systems, this species has recently gained attention in South Korean aquaculture. Compared to *A. japonica*, *A. marmorata* is more cost-effective to produce and less reliant on expensive seed sources, further enhancing its commercial appeal [17,18].

Chinese cabbage (*Brassica rapa* L.) is known for its high commercial value, rapid growth, and high nutritional content [19,20]. Under nutrient-rich aquaponic conditions, *B. rapa* exhibits vigorous vegetative development, producing up to 11 leaves and reaching 12 cm in height within four weeks [21]. It also plays a vital role in improving water quality by absorbing harmful nitrogenous compounds such as ammonia and nitrite, converting them into less toxic nitrates [22]. Furthermore, co-cultivation of *B. rapa* with fish species such as common carp has been shown to enhance fish growth and survival rates compared to systems without plants [23]. However, excessive nutrient accumulation at higher fish densities can lead to plant stress or reduced nutrient uptake [24], highlighting the importance of optimal density management.

This study is the first to establish an indoor media-based aquaponics system combining *A. marmorata* and *B. rapa* var. *pekinensis*, with a primary focus on eel culture under different stocking densities. Here, we hypothesized that moderate stocking densities would optimize the balance between eel growth and water quality, while extreme densities (either too low or too high) may result in suboptimal outcomes due to underutilization or overloading of system resources. The objective of this study is to evaluate the effects of three stocking densities of *A. marmorata* on growth performance, proximate composition, and water quality parameters in a media-based indoor aquaponics system integrated with *B. rapa* var. *pekinensis*, aiming to identify the most efficient stocking density for maximizing productivity and maintaining environmentally optimal water quality in a recirculating aquaculture system.

## 2. Materials and Methods

### 2.1. Experimental Design

**Figure 1:**
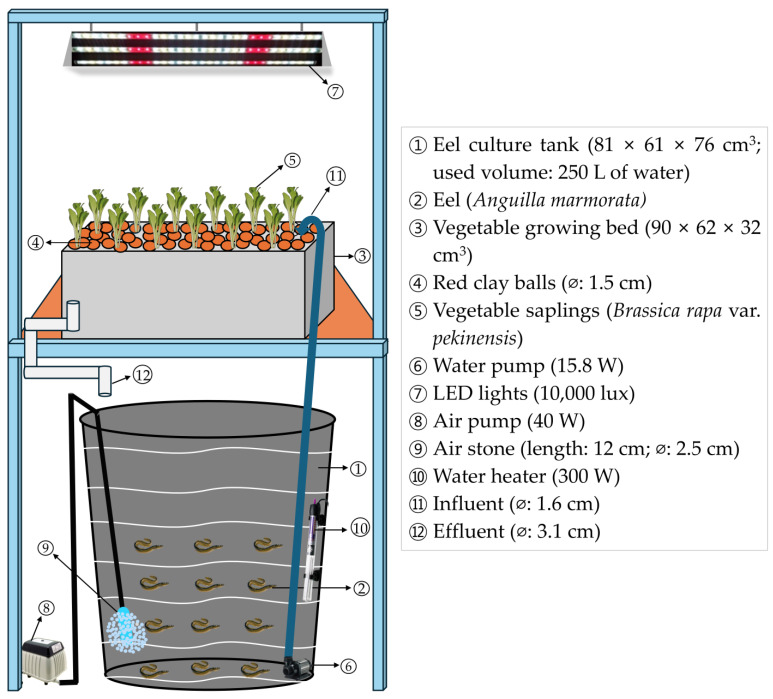
Conceptual representation of the media-based indoor aquaponics setup used in this study.

The study was conducted over 8 weeks (56 days), from 27 September–22 November 2023, at the College of Fisheries and Ocean Sciences, Chonnam National University, Yeosu-si, Jeollanam-do, Republic of Korea (126.581316° E, 34.9860324° N), under indoor conditions. A media-based, density-dependent indoor aquaponics system was employed utilizing three stocking density treatments: 10, 15, and 20 eels 250 L^−1^ of water, designated as T_1_, T_2_, and T_3_, respectively. Each treatment was replicated three times. These treatments created intentional differences in initial biomass among tanks. Individual eels were size-matched as closely as possible and randomly assigned to tanks to minimize within-treatment variance. This design enabled evaluation of how varying fish biomass loading affects nutrient dynamics, water quality, and the growth of eels. The selection of these density levels aligns with recognized practices in aquaponics, where fish biomass significantly influences outcome measures [4,25] and is supported by findings in eel-based aquaponic systems utilizing *Anguilla japonica* [26]. Each aquaponic subsystem consisted of an eel culture tank (81 × 61 × 76 cm^3^) with a total water-holding capacity of 310 L, of which 250 L was utilized. The system also included an oval-shaped Chinese cabbage cultivation bed (90 × 62 × 32 cm^3^), a siphoning tube for regulating water flow, a 300 W water heater (PhilGreen High Class Aquarium Heater PH-300, Chuangxing., Co., Hong Kong, China) for maintaining stable water temperature, and a 15.8 W submersible pump (PhilGreen Power Head Pump BT-20, Chuangxing., Co., Hong Kong, China) to transport wastewater and effluents from the eel tank to the hydroponic grow bed. Four LED plant growth lights (10,000 lux, 10 h day^−1^) were installed to provide sufficient light intensity for photosynthesis. A 3.1 cm diameter PVC drain was installed at the base of each vegetable bed to return water to the fish tank (Figure 1).

### 2.2. Eel Stocking and Rearing

One week prior to stocking, water circulation was initiated in each subsystem along with aeration and heating to stabilize dissolved oxygen (DO; mean 7.21 ± 0.02 mg L^−1^) levels and water temperature (mean 29.46 ± 0.17 °C). *A. marmorata* (average length: 43.15 ± 0.34 cm; average weight: 191.25 ± 3.70 g) were obtained from Dongwoo Fisheries Farm, Jeollanam-do, Republic of Korea. The eels were acclimatized for one week before being stocked into the T_1_, T_2_, and T_3_ tanks at densities of 10 (7.65 kg m^−3^), 15 (11.48 kg m^−3^), and 20 (15.30 kg m^−3^) eels per tank (250 L of water), respectively. As no studies have reported *A. marmorata* densities in aquaponics, these three densities were adapted from recirculating aquaculture systems to represent low, medium, and high levels [9,12,27]. The eels were maintained under 24 h darkness using a black shading net and were fed a commercial floating feed (moisture: 6.0%, crude protein: 48.0%, crude lipid: 4.0%, crude fiber: 3.0%, ash: 13.0%, calcium: 2.0%, and phosphorus: 2.7%) once daily (at 9:00 AM) at 1.75% of their body weight. Prior to feeding, the feed was moistened with tap water (1.75% of its dry weight) and shaped into dough form. Uneaten feed was siphoned out 30 to 40 min after feeding to minimize algae and fungal growth. Each eel tank was equipped with a feeding tray, an air stone (length: 12 cm; diameter: 2.5 cm), a 300 W water heater to maintain optimal temperature, and a 15.8 W submersible pump (water flow rate: 8 L min^−1^; voltage: 220 V; diameter: 1.6 cm) to transfer water and effluents from the eel tank to the vegetable bed. Aeration, heating, and filtration systems were operated continuously throughout the experimental period. No additional water was added to the fish tanks except to compensate for water loss due to evaporation and siphoning.

### 2.3. Chinese Cabbage Saplings and Growing Bed Preparation

Chinese cabbage (*B. rapa* var. *pekinensis*) saplings were purchased from a reputable plant shop in Yeosu, Republic of Korea. For the vegetable beds, nine rubber oval containers (90 × 62 × 32 cm^3^) were used. Each container was equipped with a PVC drainage outlet (diameter: 3.1 cm) connected to the eel tank. Prior to planting, all beds were thoroughly cleaned and dried under sunlight. Thoroughly cleaned red clay balls, with an average diameter of 1.5 cm, served as the growing medium, ensuring adequate root support and providing an extensive surface area for microbial colonization, which enhances nitrification [9,27]. No separate clarifier or biofilter was used, as the system was designed to evaluate its self-sufficiency in maintaining water quality and supporting eel growth performance. In each bed, eight saplings were planted, with an average initial stem length of 11.33 ± 0.42 cm, and one crop cycle was carried out for this study. Saplings were spaced 15 cm apart to allow adequate place for growth. To ensure consistent light conditions, four sets of LED grow lights (Plant LED Bar, NINE S Co., Chuncheon-si, Republic of Korea) were installed above each vegetable bed and operated for 10 h day^−1^ throughout the experimental period.

### 2.4. Initiation of Aquaponics

The experiment began after the vegetable-growing beds and eel culture tanks were fully set up. As noted by Roy et al. [24] and Nadia et al. [28], an initial “lag phase” was observed, during which nitrifying bacteria became established in the plant beds, enhancing nutrient cycling and supporting plant growth. Throughout the experimental period, dried leaves were regularly removed from the vegetable beds to maintain efficient system performance and prevent blockages or nutrient imbalances.

### 2.5. Sampling and Harvesting of Eel

Measurements of all individual eels in each tank were conducted at the beginning, middle, and end of the 8-week experimental period. Following a 24 h fasting period, all individual eels were collected from the tanks using a scoop net and transferred into a 50-L bucket. They were then anesthetized with 2-phenoxyethanol (0.1 mL L^−1^) following the protocol outlined by Tan et al. [12]. Total body length and body weight were measured using a measuring scale and an electronic balance (PAG 2102, OHAUS Co., Parsippany, NJ, USA), respectively. Growth parameters, including length gain, percent length gain (%), weight gain (WG), percent weight gain (% WG), specific growth rate (SGR, % day^−1^), feed conversion ratio (FCR), survival rate (SR, %), and production (kg m^−3^), were evaluated as described by Salama et al. [29]. Additional performance metrics, including daily weight gain (DWG) and protein efficiency ratio (PER), were assessed following the method described by Tan et al. [12]. Feed efficiency (FE) and hepatosomatic index (HSI) were assessed according to Shahkar et al. [30]; feeding rate (FR) was calculated based on Luo et al. [31]; and feed intake (FI, g fish^−1^) was determined according to Aya and Garcia [32]. All equations used to calculate performance parameters for this study are as follows:

Length gain (cm) = L_2_ (cm) − L_1_ (cm)

$$\mathrm{Percent}\;\mathrm{length}\;\mathrm{gain}\;(\%)=\frac{L_{2}-L_{1}}{L_{1}}\;\times \;100$$

Weight gain (g) = W_2_ (g) − W_1_ (g)

$$\mathrm{Percent}\;\mathrm{weight}\;\mathrm{gain}\;(\%)=\frac{W_{2}-W_{1}}{W_{1}}\;\times \;100$$

$$\mathrm{Specific}\;\mathrm{growth}\;\mathrm{rate}\;(\mathrm{SGR},\;\%\;{\mathrm{day}}^{-1})=\frac{{lnW}_{2}-{lnW}_{1}}{t(day)}\;\times \;100$$

$$\mathrm{Daily}\;\mathrm{weight}\;\mathrm{gain}\;(\mathrm{DWG},\;g\;{\mathrm{day}}^{-1})=\frac{W_{2}-W_{1}}{t(day)}$$

$$\mathrm{Feed}\;\mathrm{conversion}\;\mathrm{ratio}\;(\mathrm{FCR})=\frac{TF(g)}{Mean\;weight\;gain(g)}$$

$$\mathrm{Feed}\;\mathrm{efficiency}\;(\mathrm{FE},\;\%)=\frac{Mean\;weight\;gain(g)}{TF(g)}\;\times \;100$$

$$\mathrm{Protein}\;\mathrm{efficiency}\;\mathrm{ratio}\;(\mathrm{PER},\;\%)=\frac{Mean\;weight\;gain(g)}{TF\left(g\right)\times Feed\;protein(\%)}\;\times \;100$$

$$\mathrm{Feeding}\;\mathrm{rate}\;(\mathrm{FR},\;\%\;{\mathrm{day}}^{-1})=\frac{TF(g)}{{[(W}_{2}+W_{1})\times n\times t/2]}\;\times \;100$$

$$\mathrm{Feed}\;\mathrm{intake}\;(\mathrm{FI},\;g\;{\mathrm{fish}}^{-1})=\frac{TF(g)}{No.of\;fish\;at\;harvest}$$

$$\mathrm{Hepatosomatic}\;\mathrm{index}\;(\mathrm{HSI},\;\%)=\frac{Liver\;weight(g)}{Body\;weight(g)}\;\times \;100$$

$$\mathrm{Survival}\;\mathrm{rate}\;(\mathrm{SR},\;\%)=\frac{No.of\;fish\;harvested}{No.of\;fish\;stocked}\;\times \;100$$

Eel production = No. of fish caught × Mean increased weight (g)

where L_1_ and L_2_ are the initial and final body lengths (cm); W_1_ and W_2_ are the initial and final body weights (g) of the eels; t is the duration of the experiment (56 days); TF is the total amount of feed (g) given during the experimental duration; and n is the number of initial eels in each tank.

### 2.6. Water Sampling of the Eel Tank

Throughout the experiment, water quality parameters were regularly monitored. A YSI meter (ProDSS, YSI Inc., Yellow Springs, OH, USA) was used to measure water temperature, pH, and DO. Electrical conductivity (EC) and total dissolved solids (TDS) were measured weekly using an E-1 portable meter (ID944, COMS, Beijing, China). Additionally, API freshwater test kits (Mars Fishcare, Inc., USA) were used weekly to assess total ammonia nitrogen (TAN), nitrite (NO_2_), and nitrate (NO_3_) concentrations. Phosphate (PO_4_) concentrations in the eel tanks were also measured weekly at the Sienna Marine Research Institute. At the end of the experiment, the concentrations of potassium (K), calcium (Ca), magnesium (Mg), and sulfur (S) were analyzed at the Sienna Marine Research Institute in Yeosu-si, Republic of Korea.

### 2.7. A. marmorata Proximate Composition Analysis

The moisture, ash, crude protein, and crude lipid contents of *A. marmorata* were analyzed following standard techniques outlined by the AOAC [33] (Association of Official Analytical Chemists). Three eels were randomly selected from each treatment group and whole eels were used for this proximate composition analysis. An automatic solvent extractor (SER 158/6, VELP Scientifica, Usmate, Italy) was used to determine moisture content by desiccation at 105 °C, ash content by incineration at 550 °C, and crude lipid content via Soxhlet extraction. Crude protein content was calculated by multiplying the total nitrogen content by 6.25 and was measured using an elemental analyzer (Vario MAX cube, Elementar Analysensysteme GmbH, Langenselbold, Hesse, Germany).

### 2.8. Sampling and Harvesting of B. rapa var. pekinensis

The shoot length of the plant was measured (initial and final) from the root collar to the tip of the main stem using a plastic ruler. In addition, the number of leaves and chlorophyll content (SPAD-502PLUS, Konica Minolta, Inc., Tokyo, Japan) were recorded every two weeks. At the final day of the experiment, plants were uprooted from each bed, and measurements of total plant length, final shoot length, and final root length were recorded.

### 2.9. Data Analysis

All individual eels in each tank were measured, and the tank mean was used as the experimental unit for statistical analysis; three replicate tanks per treatment were considered as true replicates. Water quality parameters were measured across all tanks, with each tank considered a single replicate, whereas plant parameters were measured across all vegetable beds, with each bed represented as a single replicate. Descriptive statistics were presented as mean ± standard deviation (SD), representing variability among individuals within tanks for fish and among tanks and beds for water and plant parameters, respectively. Comparisons among the three treatments were performed using one-way ANOVA followed by Duncan’s multiple range test (DMRT) [34], with significance at *p* < 0.05. Although each treatment included only three replicate tanks, formal tests for normality and homogeneity have limited power; data distributions were inspected, and the use of ANOVA follows common practices in aquaponics research [27]. All statistical analyses were performed using SPSS (version 27.0), and graphs were generated using GraphPad Prism (version 8.0.2).

## 3. Results

### 3.1. Water Quality Parameters

**Figure 2:**
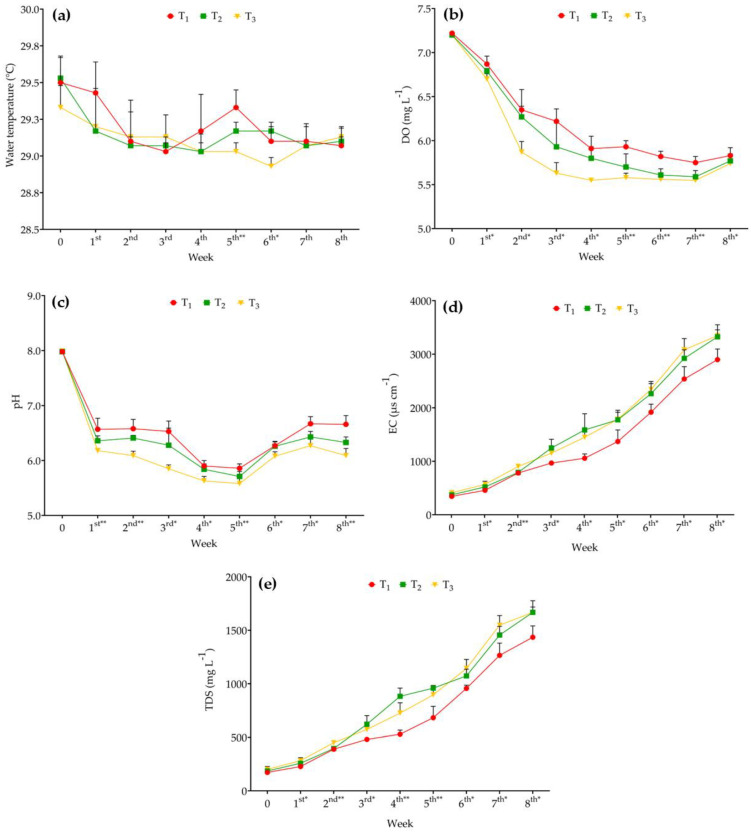
(**a**) Water temperature, (**b**) DO (dissolved oxygen), (**c**) pH, (**d**) EC (electrical conductivity), and (**e**) TDS (total dissolved solids) in T_1_ (10 eels 250 L^−1^ of water), T_2_ (15 eels 250 L^−1^ of water), and T_3_ (20 eels 250 L^−1^ of water) measured throughout the experimental period. Different colored symbols represent measured values (mean ± SD based on three replicate tanks per treatment) recorded during different sampling weeks. * indicates significance at *p* ≤ 0.05, and ** indicates significance at *p* ≤ 0.01 among the treatments.

Water temperature in the eel tanks ranged from 28.93 °C to 29.53 °C throughout the experiment. Significant differences among the treatments were observed only during the fifth (*p* < 0.01) and sixth weeks (*p* < 0.05), with no other significant variations (*p* > 0.05) observed during the other sampling weeks (Figure 2a). The DO levels were recorded as follows: T_1_: 5.75–7.22 mg L^−1^; T_2_: 5.59–7.20 mg L^−1^; and T_3_: 5.55–7.20 mg L^−1^. Significant differences (*p* < 0.05) in DO were observed among the treatments across different sampling weeks (Figure 2b). Furthermore, significant differences in pH (*p* < 0.05) were observed among all treatments from the first to the eighth week of the sampling period (Figure 2c).

EC and TDS also showed significant differences (*p* < 0.05) among treatments during every sampling week. By the final sampling period (eighth week), the highest EC value was recorded in T_3_ (3346.67 ± 108.56 µs cm^−1^), followed by T_2_ (3324.67 ± 224.89 µs cm^−1^) and T_1_ (2898.33 ± 199.26 µs cm^−1^) (Figure 2d). Additionally, at the end of the experiment, T_2_ exhibited the highest TDS value (1667.67 ± 109.49 mg L^−1^), whereas T_1_ presented the lowest value (1435.33 ± 106.05 mg L^−1^) (Figure 2e).

**Figure 3:**
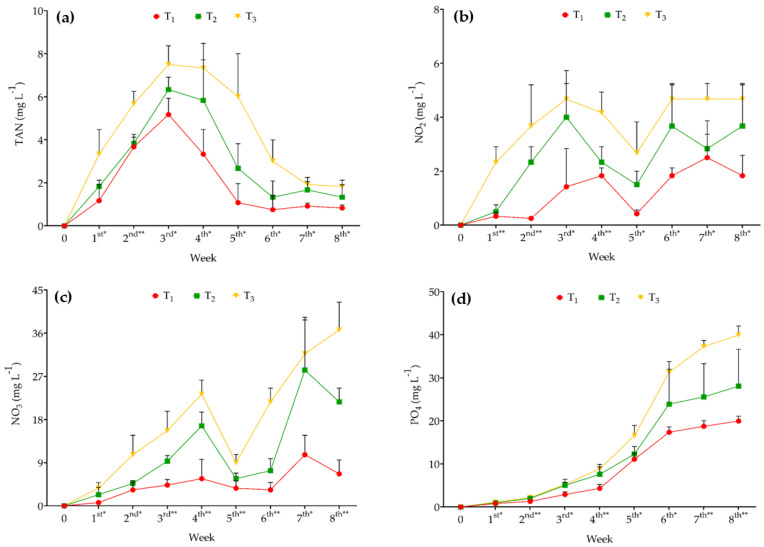
(**a**) TAN (total ammonia nitrogen), (**b**) NO_2_ (nitrite), (**c**) NO_3_ (nitrate), and (**d**) PO_4_ (phosphate) in T_1_ (10 eels 250 L^−1^ of water), T_2_ (15 eels 250 L^−1^ of water), and T_3_ (20 eels 250 L^−1^ of water) measured throughout the experimental period. Different colored symbols represent measured values (mean ± SD based on three replicate tanks per treatment) recorded during different sampling weeks. * indicates significance at *p* ≤ 0.05, and ** indicates significance at *p* ≤ 0.01 among the treatments.

The mean TAN concentration in T_3_ was 32.02% and 53.69% higher than in T_2_ and T_1_, respectively, with significant differences among treatments observed during the sampling weeks (*p* < 0.05; Figure 3a). NO_2_ concentrations fluctuated throughout the experimental period, with the highest mean values recorded at different time points (T_1_: 2.50 ± 0.87 mg L^−1^ on the 7th week; T_2_: 4.00 ± 1.73 mg L^−1^ on the 3rd week; and T_3_: 4.67 ± 0.58 mg L^−1^ on the 3rd, 6th, 7th, and 8th weeks), showing significant differences among treatments (*p* < 0.05; Figure 3b). Similarly, NO_3_ concentrations varied over time, reaching the highest recorded values in the 8th week: 36.67 ± 5.77 mg L^−1^ in T_3_, followed by 21.67 ± 2.89 mg L^−1^ in T_2_ and 6.67 ± 2.89 mg L^−1^ in T_1_. PO_4_ concentrations also fluctuated throughout the experiment, with the highest mean value observed in T_3_ (39.92 ± 2.10 mg L^−1^) and the lowest in T_1_ (19.95 ± 1.13 mg L^−1^) at the end of the trial. Significant differences among all treatments in NO_3_ and PO_4_ concentrations were found during the sampling weeks, except at week 0 (Figure 3c,d).

**Figure 4:**
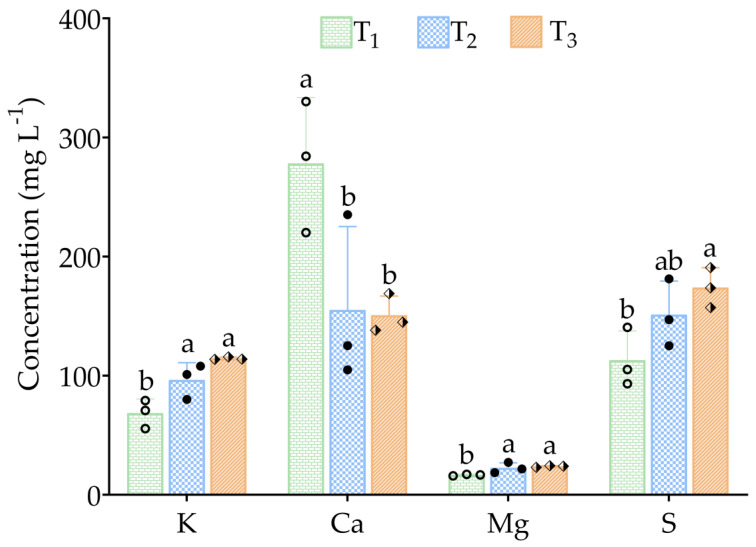
Mean concentrations (mg L^−1^) of minerals (K = potassium; Ca = calcium; Mg = magnesium; and S = sulfur) in tank water of a media-based indoor aquaponics. Mean values (± SD) were calculated based on three replicate tanks per treatment: T_1_ = 10 eels 250 L^−1^ of water; T_2_ = 15 eels 250 L^−1^ of water; and T_3_ = 20 eels 250 L^−1^ at the end of this experiment. Different symbols in the bars represent the mean values of the replicate tanks. Bars with different letters indicate significant differences (*p* ≤ 0.05) based on Duncan’s multiple range test (DMRT).

At the end of the experiment, T_3_ exhibited the highest concentrations of K (114.47 ± 1.16 mg L^−1^; *p* < 0.01), Mg (23.83 ± 0.67 mg L^−1^; *p* < 0.05), and S (173.90 ± 16.80 mg L^−1^; *p* < 0.05) (Figure 4). In contrast, Ca concentrations were 278.20 ± 55.30, 155.13 ± 70.09, 150.73 ± 16.18 mg L^−1^ in T_1_, T_2_, and T_3_, respectively. The differences in Ca concentrations among the treatments were highly significant, with T_2_ and T_3_ showing the lowest values (*p* < 0.05; Figure 4).

### 3.2. Growth Performance of A. marmorata

**Figure 5:**
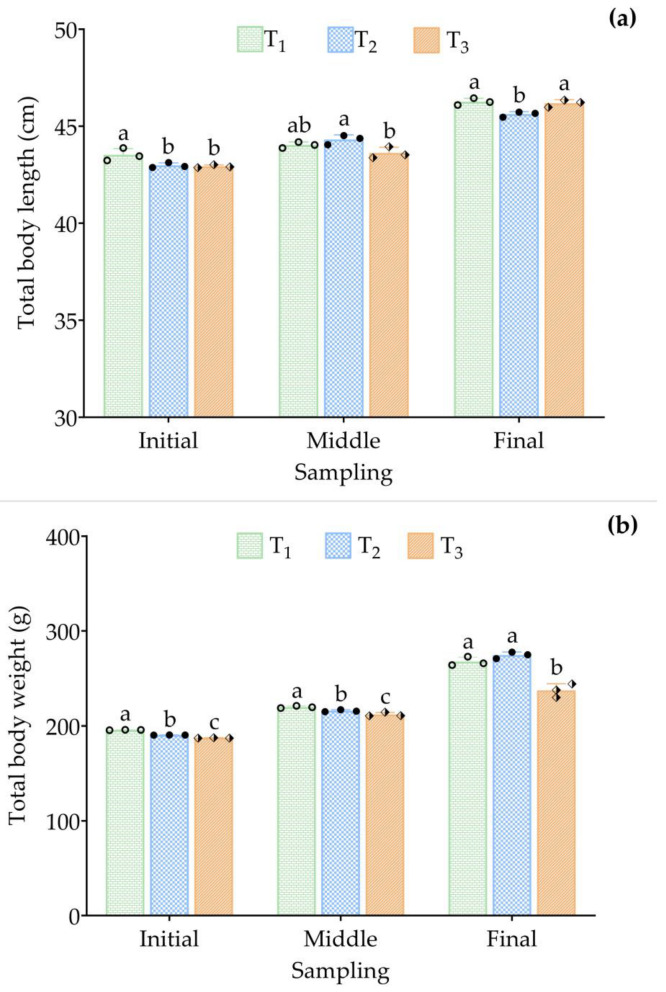
(**a**) Total body length and (**b**) total body weight of *A. marmorata* during the initial, middle, and final sampling periods. Mean values (± SD) were calculated based on three replicate tanks per treatment: T_1_ = 10 eels 250 L^−1^ of water; T_2_ = 15 eels 250 L^−1^ of water; and T_3_ = 20 eels 250 L^−1^ of water. Different symbols in the bars represent the mean values of the replicate tanks. Bars with different letters indicate significant differences (*p* ≤ 0.05) based on Duncan’s multiple range test (DMRT).

At week 8, T_1_ and T_2_ exhibited the highest mean final body length (46.27 ± 0.18 cm; *p* < 0.05) and body weight (274.44 ± 3.41 g; *p* < 0.05), respectively (Figure 5a,b), compared to T_3_. T_2_ showed significantly (*p* < 0.01) higher weight gain (83.98 ± 3.54 g), percent weight gain (44.09 ± 1.89%), and specific growth rate (0.65 ± 0.02% day^−1^) compared to T_3_, which had the lowest values (49.78 ± 6.97 g; 26.56 ± 3.69%; 0.42 ± 0.05% day^−1^, respectively) (Table 1). Daily weight gain in T_2_ was 14.67% higher than in T_1_ and 40.67% higher than in T_3_ (*p* < 0.01). Feed conversion ratio was lowest (2.52 ± 0.11), and feed efficiency was highest in T_2_ (39.73 ± 1.66%) compared to the other treatments. However, T_1_ had a significantly higher protein efficiency ratio (6.14 ± 0.41%; *p* < 0.01) and feeding rate (1.88 ± 0.03% day^−1^; *p* < 0.01) than both T_2_ and T_3_ (Table 1). Mean feed intake was highest in T_1_ (243.39 ± 0.68 g fish^−1^) and lowest in T_3_ (194.80 ± 0.97 g fish^−1^), while T_3_ had the highest hepatosomatic index (1.60 ± 0.06%). All treatments had 100% survival rates. Notably, eel production was significantly higher (*p* < 0.01) in T_3_ (18.98 ± 0.57 kg m^−3^) compared to T_2_ (16.47 ± 0.21 kg m^−3^) and T_1_ (10.71 ± 0.19 kg m^−3^) (Table 1).

### 3.3. Proximate composition of A. marmorata

All proximate parameters (moisture, crude protein, crude lipid, and ash) differed significantly among treatments (*p* < 0.01) (Table 2). Moisture content was significantly higher in T_3_ (67.97 ± 0.69%) compared to T_2_ (64.37 ± 0.85%) and T_1_ (62.14 ± 0.51%). Crude protein increased progressively from T_1_ (16.77 ± 0.61%) to T_2_ (17.53 ± 0.31%) and T_3_ (18.24 ± 0.18%). Moreover, the highest crude lipid (9.26 ± 0.15%) and ash (2.42 ± 0.12%) contents were observed in T_1_ and T_2_, respectively (Table 2).

### 3.4. Growth Performance of B. rapa var. pekinensis

**Figure 6:**
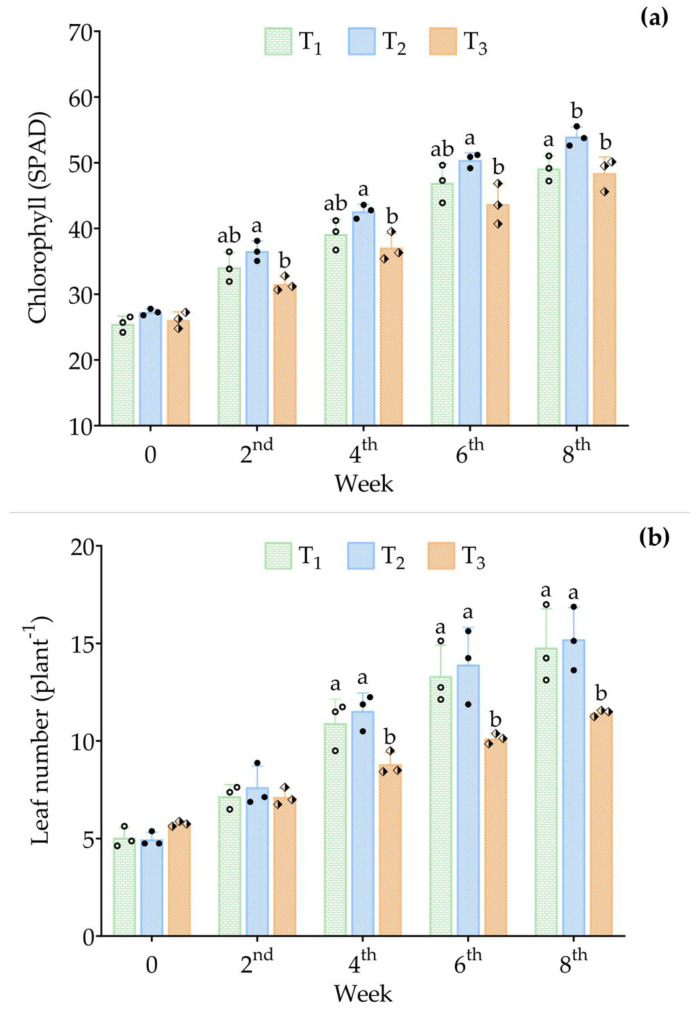
(**a**) Chlorophyll (SPAD) and (**b**) leaf number (plant^−1^) of *B. rapa* var. *pekinensis* at different sampling weeks throughout the experimental period. Mean values (±SD) were calculated based on three replicate tanks per treatment: T_1_ = 10 eels 250 L^−1^ of water; T_2_ = 15 eels 250 L^−1^ of water; and T_3_ = 20 eels 250 L^−1^ of water. Different symbols in the bars represent the mean values of the replicate vegetable beds. Bars with different letters indicate significant differences (*p* ≤ 0.05) based on Duncan’s multiple range test (DMRT). The absence of letters in the bars denotes no significant difference (*p* > 0.05).

At the beginning of the experiment, the total plant length, shoot length, and root length of *B. rapa* var. *pekinensis* saplings were similar among all treatments (*p* > 0.05) (Table 3). At the end of the experiment, the highest plant length (29.64 ± 1.67 cm) was observed in T_2_, with significant differences (*p* < 0.05). Although T_2_ also exhibited the highest values for final shoot length and final root length, these differences were not statistically significant (*p* > 0.05). The highest mean chlorophyll content was recorded in T_2_, with significant differences (*p* ≤ 0.05) among treatments across sampling weeks, except at week 0 (Figure 6a). Similarly, T_2_ consistently showed the highest mean leaf number compared to T_1_ and T_3_ among sampling weeks, with significant differences (*p* ≤ 0.05), except at week 0 and week 2 (Figure 6b).

## 4. Discussion

This study is the first to establish an indoor aquaponics system assessing the impact of stocking densities of giant mottled eel (*A. marmorata*) co-cultivated with Chinese cabbage (*B. rapa* var. *pekinensis*). Achieving a sustainable balance between the economic objectives of intensive eel farming and the environmental concerns related to increased freshwater discharge is essential. The findings of this study provide valuable insights into the sustainability of giant mottled eel culture in indoor aquaponics by identifying the optimal stocking density to maximize system efficiency and biomass production. This discussion relates the findings within the context of previous studies and explores their implications for scaling and improving indoor aquaponic systems to promote sustainable food production.

### 4.1. Water Quality Responses to Varying Stocking Densities

Water quality is a critical factor influencing the growth and development of aquatic organisms and is essential for the successful operation of aquaponic systems. Key parameters include adequate tank aeration, maintaining appropriate pH levels, effective biological conversion of harmful TAN and ammonia (NH_3_) to beneficial nitrate, and efficient plant uptake of dissolved nutrients [35,36]. Across treatments, water quality largely remained within optimal ranges for most fish species, even at higher stocking densities [37]. Although the measured parameters aligned well with values reported in prior studies [38,39,40], stocking density significantly affected several parameters. Specifically, increased stocking density led to elevated TAN and nitrite (NO_2_) concentrations, underscoring the need for careful monitoring and management to maintain fish health. Potential adverse effects of stocking density on water quality in aquaponics can be mitigated by proper system design, regular maintenance, and consistent monitoring of key water quality indicators. Further research is needed to better understand nutrient cycling and ecosystem balance for optimizing aquaculture practices.

Water temperature in eel tanks remained stable (28.93–29.53 °C) throughout the experiment, with no significant differences among treatments, indicating that stocking density did not affect this parameter. The temperature range was maintained consistently within the recommended levels for warm-water fish (22–32 °C) and eel growth (25–32 °C), as reported by Yildiz et al. [11] and Roosta and Hamidpour [41]. Minor temperature fluctuations observed during the study were likely influenced by water circulation through the inlet–outlet system and ambient room temperature variations [42].

DO is vital for fish physiology and survival and essential for nitrification processes. DO levels inversely correlate with water temperature [43]. Maintaining DO levels above 5 mg L^−1^ is critical for fish health and growth, whereas levels below 2 mg L^−1^ can inhibit nitrification by suppressing key microbial functions [44,45]. Higher stocking densities can reduce DO levels due to increased fish metabolic demand and accumulation of organic matter, which raises microbial oxygen consumption during oxidation [46,47]. Throughout the experiment, dissolved oxygen levels remained within the acceptable range, measured 5.75–7.22 mg L^−1^ in T_1_, 5.59–7.20 mg L^−1^ in T_2_, and 5.55–7.20 mg L^−1^ in T_3_.

pH significantly influences the health and productivity of aquatic organisms, plants, and microbial communities in aquaponic systems. In soilless cultivation, water pH regulates nutrient bioavailability and uptake, which is essential for plant growth [36]. A decrease in pH was observed with increasing fish stocking density, consistent with findings by Goddek et al. [4] and Kloas et al. [48]. This continuous fluctuation in pH across all treatments over time likely resulted from carbon dioxide (CO_2_) emissions during fish respiration and bacterial conversion processes, which increase hydrogen ion (H^+^) concentration, lowering pH [49]. Additionally, the conversion of NH_3_ under nutrient-rich conditions can contribute to further pH declines [50]. According to Zou et al. [51], different fish species respond uniquely to pH variations, highlighting the importance of maintaining pH stability to ensure system resilience and biological integrity. In this study, the pH was maintained between 5.58 and 7.99 across treatments, primarily within the ideal range (5.5–7.5) recommended for plant cultivation by Somerville et al. [9]. In this study, occasional pH levels dropped below 6. Despite these fluctuations, no mortality or visible skin damage occurred in the eels, suggesting tolerance to pH variations. Anguillid eels typically prefer near-neutral conditions (6.5–8.5) [52], but adult eels can tolerate short-term acidic environments. For instance, longfin eels (*A. dieffenbachii*) maintained 100% survival at pH 4.5 for 14 days, although no species survived below pH 4 [53]. Even though no external skin lesions or abnormal behavior were observed, pH fluctuations may have imposed sublethal stress, as acidic conditions can affect mucosal integrity and increase physiological load. Despite the absence of visible stress, sublethal effects such as impaired mucosal integrity or increased physiological load may have occurred. No external pH adjustments or nutrient supplementation were performed; all nutrients were derived from the fish feed. The observed pH decline was likely caused by nitrification and plant uptake of basic ions, which can reduce carbonate alkalinity over time. Alkalinity was not measured in this study, which limits our ability to fully interpret the stability of water chemistry. Aquaculture guidelines emphasize the importance of pH stability to ensure welfare and control water quality [27,54,55,56]. Nevertheless, maintaining a stable pH is recommended to minimize potential welfare risks and optimize growth conditions in indoor aquaponics systems. Future studies should include alkalinity monitoring and consider pH management strategies to ensure system stability and optimize growth conditions in media-based aquaponics systems.

EC in aquaponic systems reflects the concentration of ions relevant to plant nutrient uptake [57], while TDS represents the overall concentrations of dissolved substances affecting both aquatic and plant components of the system [58]. Maintaining EC and TDS within optimal ranges is essential for ensuring nutrient balance and system stability. Elevated levels can lead to osmotic stress in fish and hinder plant growth, while low levels may indicate a deficiency of essential nutrients. Thus, maintaining these parameters within appropriate limits is crucial for sustaining the integration of fish and plants and for maximizing system productivity. To our knowledge, no published data is currently available on EC and TDS values specifically for *A. marmorata* in aquaponic systems, making direct comparisons with existing literature difficult. In this study, the highest EC (3346 µS cm^−1^) and TDS (1667 mg L^−1^) measured correspond to a salinity of approximately 1.7‰ [59], which falls within the low brackish water range. This observation is consistent with the salinity tolerance of *A. marmorata*, which is known to survive in salinities ranging from 0–17‰ and grows optimally at 10–17‰ [60,61]. These findings support the suitability of *A. marmorata* for low salinity aquaponic systems. Higher stocking densities in an aquaponics system increase metabolic waste and feed input, leading to the accumulation of dissolved ions and solids and, consequently, elevated EC and TDS levels [62,63], as observed in T_2_ and T_3_ in this study. If not adequately managed, these elevated levels can impair water quality, disrupt nutrient dynamics, and reduce the overall efficiency of the system.

In fish culture systems, fish waste and uneaten feed are the primary sources of TAN [64]. Consequently, lower fish stocking densities generally result in reduced TAN levels in the water [47], as was observed in this study. The significantly higher concentrations of TAN, NO_2_, and NO_3_ in T_3_ indicate that the elevated stocking density had a notable impact on nutrient cycling. These effects are likely due to increased feed input and metabolic waste, which accelerate the production and accumulation of organic matter and nitrogenous compounds. Moreover, these inputs enhance the ammonification and nitrification processes, leading to increased concentrations of TAN and its oxidized forms, thereby affecting overall nitrogen dynamics in the system [24,55,65,66]. Similarly, the medium-density treatment (T_2_) also exhibited significantly elevated nutrient levels compared to the low-density treatment (T_1_). These findings are consistent with previous studies by Ani et al. [2] and Rahmatullah et al. [67], who reported increases in NO_3_ concentrations with increasing fish stocking densities and feed inputs. While plants can directly absorb ammonium (NH_4_), nitrate (NO_3_) must first be reduced before uptake [49]. NO_3_ is generally regarded as non-toxic to fish under normal conditions but may pose risks if it accumulates to excessively high levels [68]. Therefore, maintaining optimal nutrient concentrations is essential for supporting the health and productivity of both fish and plants in aquaponic systems.

PO_4_ levels also require close monitoring, as excessive accumulation can impair water quality, disrupt microbial processes within the nitrogen cycle, and destabilize system balance [69]. In this study, the highest PO_4_ concentration (36.59 mg L^−1^) was observed in T_3_, which exceeded the commonly recommended range for aquaponic systems (1–10 mg L^−1^) [25,27]. This result can be attributed to increased feed input at higher stocking densities, as fish feed is the primary phosphorus source in recirculating and aquaponics systems [65,66]. When feed-derived phosphorus and excretory waste accumulate faster than plants or microbial processes assimilate them, PO_4_ levels can exceed recommended thresholds, potentially impairing water quality and system stability [27,55]. Although these elevated concentrations could promote vigorous plant growth if nutrient uptake is well-regulated, maintaining appropriate stocking densities remains crucial to avoid nutrient overload and preserve system stability [48,62].

K, Ca, Mg, and S are essential nutrients that play crucial roles in plant growth and metabolism within aquaponic systems, with tailored fish feeds enhancing plant biomass and preventing deficiencies. Maintaining optimal levels of these elements is essential for supporting the health and productivity of both fish and plants in aquaponics systems. In media-based systems, typical optimal ranges for plant essential macronutrients are K, 50–150 mg L^−1^; Ca, 40–80 mg L^−1^; Mg, 20–50 mg L^−1^; and S, 20–50 mg L^−1^, depending on species (both fish and plant) and system design [27,55,65]. Although the suggested ranges of those conditions are not fully matched with this study design, in terms of optimal ranges, K and Mg were generally within or near commonly reported optimal ranges for aquaponic systems. In contrast, the Ca and S concentrations exceeded the typical optimal ranges, likely due to accumulation from fish feed and minerals in the water source. K supports photosynthesis, Ca is essential for cell structure and membrane integrity, Mg is a key component of chlorophyll, and S is involved in amino acid synthesis [69,70,71,72]. Moreover, previous studies by Roy et al. [24] and Maucieri et al. [73] have revealed significant increases in the concentrations of K, Ca, Mg, and S with rising fish stocking densities. The findings of the current study largely support these observations, except Ca, which showed a decrease as stocking density increased. This decline in Ca concentration may be due to heightened competition among fish for available calcium at higher densities, potentially reducing individual uptake. Additionally, increased organic matter and microbial activity associated with higher stocking densities may influence Ca dynamics in the system, either by promoting Ca precipitation or uptake by other organisms, thus reducing its availability in the water column.

### 4.2. Growth Responses of A. marmorata to Different Stocking Densities

Stocking density is a critical factor influencing key production parameters in commercial aquaculture systems [74]. Among these, growth is one of the most extensively studied and reliable indicators, as it reflects both physiological performance and stress levels in fish [12]. Chronic stress caused by high stocking densities can negatively affect fish growth and development, though the severity of these impacts varies depending on the culture system, species-specific responses, and developmental stages [75,76].

Moreover, the observed differences in initial fish biomass across treatments were not arbitrary but integral to the experimental design, which aimed to assess density-dependent interactions between *A. marmorata* and *B. rapa* var. *pekinensis*. Stocking density and fish-to-plant biomass ratios are widely considered critical determinants of nutrient cycling, water quality, and plant growth in aquaponic systems [4,25]. Notably, experiments using *A. japonica* in hybrid biofloc–aquaponic setups demonstrated that eel biomass loading significantly influenced leafy vegetable productivity and water nutrient dynamics [26]. 

Survival rate is another critical indicator of a species’ adaptation to its environment. In this study, survival remained at 100% throughout the entire experimental period. This study’s result is consistent with previous reports for species such as the giant mottled eel (*Anguilla marmorata*), rainbow trout (*Oncorhynchus mykiss*), sea bass (*Dicentrarchus labrax*), red porgy (*Pagrus pagrus*), and Nile tilapia (*Oreochromis niloticus*) [77,78,79]. Although these cited studies were conducted under different stocking densities and experimental conditions, they provide a general context for survival rates under supporting densities. In contrast, Ashley [74] and Ellis et al. [80] reported injuries and mortalities at higher stocking densities in other fish species. The decrease in the SGR of eels under high stocking density in this study may be attributed to increased competition for space and feed [24,73].

At the end of the trial, the medium-density group (T_2_) exhibited significantly better growth performance in terms of WG, %WG, DWG, and FE. However, despite lower individual growth rates, total eel production was higher in T_3_ due to the larger number of fish. Similar trends have been reported in Japanese flounder [81], Nile tilapia [82,83], red tilapia [84], channel catfish [85], and juvenile pirarucu [86]. Additionally, T_1_ exhibited better individual growth parameters than T_3_, suggesting that lower stocking densities do not negatively affect *A. marmorata* growth, while higher densities may reduce growth due to increased competition and aggression, limiting feed access and raising the energetic cost of feeding [87]. Considering both system efficiency and fish growth, a stocking density of 15 eels 250 L^−1^ of water (T_2_) appears to be optimal for indoor media-based aquaponics systems. Future studies should explore the performance of *A. marmorata* at densities exceeding 15 eels per 250 L^−1^ of water under modified aquaponic conditions.

### 4.3. Effect of Different Stocking Densities on the Proximate Composition of A. marmorata

Body composition is a key indicator of fish health and quality [84]. However, there is limited research on how different stocking densities affect the body composition of *A. marmorata*. In this study, the moisture content ranged from 62.14% (T_1_) to 67.97% (T_3_), crude protein 16.77% to 18.24%, crude lipid 4.94% to 9.26%, and ash content 1.93% to 2.42%. These results are more or less similar to those reported for the giant mottled eel by Tan et al. [12], who observed moisture levels of 65–68%, protein 17–19%, lipid 4–8%, and ash 2.0–2.3% under varying culture conditions (recirculating aquaculture system). Compared to monosex Nile tilapia (70–75% moisture, 15–18% protein, 3–6% lipid, and 1–2% ash) [2], *A. marmorata* of this study exhibited slightly lower moisture but higher lipid content at low stocking densities (T_1_). In contrast, rainbow trout typically contain 8–12% lipid and 68–70% moisture [88], showing a much higher lipid content than the reduced levels observed in *A. marmorata* at high stocking density (T_3_) in this study. These differences suggest species-specific responses to stocking density, with *A. marmorata* maintaining relatively high protein, moisture, and ash content but showing a marked reduction in lipid at higher densities. Notably, the medium-density group (T_2_) exhibited significantly higher protein and ash content compared to the low-density group (T_1_), indicating possible mineral retention adaptations under moderate stocking densities.

### 4.4. Growth Performance of B. rapa var. pekinensis to Different Stocking Densities

This study demonstrated that fish stocking density significantly affected the growth of *B. rapa* var. *pekinensis* in aquaponics. While initial traits were uniform, at the end of the experiment, plants in T_2_ (15 eels 250 L^−1^) achieved the highest plant length, chlorophyll content, and leaf number. Moderate density is likely to ensure balanced nutrient supply and efficient nitrogen assimilation, enhancing photosynthesis and leaf development. Similar findings have been reported in aquaponics, where intermediate fish stocking densities optimize nutrient availability and plant performance, while lower stocking densities cause deficiencies and higher densities lead to metabolite accumulation, such as ammonia [27,66,89,90].

The higher leaf number and chlorophyll content in T_2_ are particularly important for leafy vegetables, as they directly contribute to yield. Reduced performance in T_3_ suggests that excessive stocking densities disrupt the balance of nutrients and microorganisms, consistent with previous studies [91,92]. It is worth noting that the system was maintained at a relatively high temperature suitable for eel culture, which may not be optimal for *B. rapa* growth and could partly explain the limited differences among shoot and root traits. Overall, the findings of this study highlight the importance of stocking density optimization in aquaponics and suggest that T_2_ provides the most favorable conditions for *B. rapa*. Future studies should expand on these results by monitoring nutrient dynamics (e.g., nitrogen species, phosphorus, and micronutrients), temperature, and water quality parameters in greater detail to establish mechanistic links between fish stocking density, nutrient availability, and plant physiological responses. Additionally, trials over longer growth cycles and across various leafy vegetables would further validate stocking density guidelines for commercial aquaponics practices.

**Figure 7:**
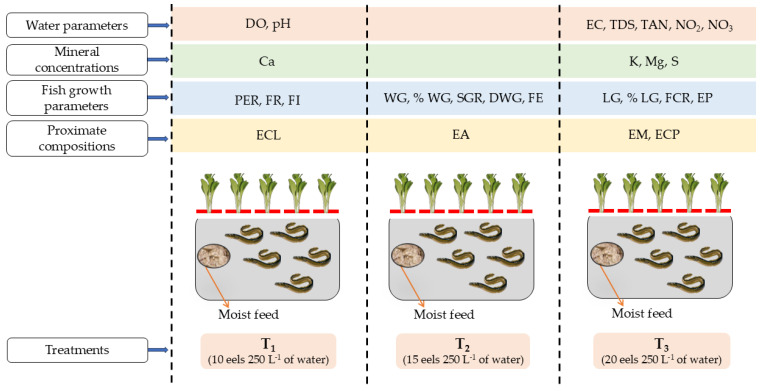
The conceptual framework of the experiment is depicted in the diagram, where the three experimental treatments (T_1_ = 10 eels 250 L^−1^ of water; T_2_ = 15 eels 250 L^−1^ of water; and T_3_ = 20 eels 250 L^−1^ of water) are outlined. Furthermore, the various colored horizontal stripes at the top of this picture represent the most significant outcomes of the key parameters in this experiment. The abbreviations used are as follows: % LG = percent length gain; % WG = percent weight gain; Ca = calcium; DO = dissolved oxygen; DWG = daily weight gain; EA = eel ash content; EC = electrical conductivity; ECL = eel crude lipid; ECP = eel crude protein; EM = eel moisture content; EP = eel production; FCR = feed conversion ratio; FE = feed efficiency; FI = feed intake; FR = feeding rate; K = potassium; LG = length gain; Mg = magnesium; NO_2_ = nitrite; NO_3_ = nitrate; PER = protein efficiency ratio; pH = potential of hydrogen; S = sulfur; SGR = specific growth rate; TAN = total ammonia nitrogen; TDS = total dissolved solids; and WG = weight gain.

Following the completion of the experiment, Figure 7 illustrates the highest significant outcomes of the key parameters of this experiment.

## 5. Conclusions

This study highlights the critical role of stocking density in aquaponic systems, particularly for *A. marmorata*. The results indicate that a moderate stocking density (15 eels 250 L^−1^ of water) offers the best balance between fish and plant growth, water quality, and overall system sustainability in small-scale aquaponic setups. Although higher stocking densities enhanced nutrient availability, they had a negative impact on fish growth. In contrast, lower densities led to underutilization of the system’s capacity and reduced overall productivity. These findings highlight the importance of carefully managing stocking density to ensure optimal fish health, water quality, and system performance. Although *A. marmorata* can tolerate short-term pH fluctuations, maintaining stable pH within the optimal range is essential to minimize stress and support welfare in future aquaponic systems.

While this study primarily focused on eel growth and water quality, preliminary observations indicated that nutrient dynamics also influenced the growth of *B. rapa* var. *pekinensis* (Chinese cabbage). Future research should simultaneously evaluate fish and plant performance for assessing the overall system productivity, nutrient dynamics, and fish–plant interactions, providing more comprehensive insights for optimizing integrated media-based aquaponics, particularly under resource-limited conditions. Importantly, these findings also provide practical implications for aquaponics systems, where balanced stocking strategies can enhance productivity, sustainability, and long-term viability of integrated farming.

## Figures and Tables

**Table 1:** Growth performance and production of *A. marmorata* in an indoor media-based aquaponics system.

Traits	T_1_	T_2_	T_3_	Significance
Initial body length (cm)	43.53 ± 0.33 ^a^	42.98 ± 0.13 ^b^	42.93 ± 0.08 ^b^	**
Middle body length (cm)	44.04 ± 0.16 ^ab^	44.32 ± 0.24 ^a^	43.62 ± 0.30 ^b^	*
Final body length (cm)	46.27 ± 0.18 ^a^	45.62 ± 0.14 ^b^	46.19 ± 0.19 ^a^	*
Length gain (cm)	2.74 ± 0.15 ^b^	2.64 ± 0.27 ^b^	3.26 ± 0.17 ^a^	*
Percent length gain (%)	6.30 ± 0.38 ^b^	6.15 ± 0.64 ^b^	7.58 ± 0.40 ^a^	*
Initial body weight (g)	195.86 ± 0.21 ^a^	190.46 ± 0.13 ^b^	187.44 ± 0.20 ^c^	**
Middle body weight (g)	219.78 ± 1.14 ^a^	215.71 ± 1.08 ^b^	211.86 ± 2.18 ^c^	**
Final body weight (g)	267.61 ± 4.68 ^a^	274.44 ± 3.41 ^a^	237.22 ± 7.16 ^b^	**
Weight gain (g)	71.75 ± 4.65 ^b^	83.98 ± 3.54 ^a^	49.78 ± 6.97 ^c^	**
Percent weight gain (%)	36.63 ± 2.37 ^b^	44.09 ± 1.89 ^a^	26.56 ± 3.69 ^c^	**
Specific growth rate (% day^−1^)	0.56 ± 0.03 ^b^	0.65 ± 0.02 ^a^	0.42 ± 0.05 ^c^	**
Daily weight gain (g day^−1^)	1.28 ± 0.08 ^b^	1.50 ± 0.07 ^a^	0.89 ± 1.26 ^c^	**
Feed conversion ratio	3.40 ± 0.22 ^a^	2.52 ± 0.11 ^b^	3.96 ± 0.56 ^a^	**
Feed efficiency (%)	29.49 ± 1.98 ^b^	39.73 ± 1.66 ^a^	25.55 ± 3.56 ^b^	**
Protein efficiency ratio (%)	6.14 ± 0.41 ^a^	5.52 ± 0.23 ^a^	2.66 ± 0.37 ^b^	**
Feeding rate (% day^−1^)	1.88 ± 0.03 ^a^	1.62 ± 0.01 ^b^	1.64 ± 0.03 ^b^	**
Feed intake (g fish^−1^)	243.39 ± 0.68 ^a^	211.35 ± 0.51 ^b^	194.80 ± 0.97 ^c^	**
Hepatosomatic index (%)	1.49 ± 0.07	1.56 ± 0.05	1.60 ± 0.06	NS
Survival rate (%)	100	100	100	-
Eel production (kg m^−3^)	10.71 ± 0.19 ^c^	16.47 ± 0.21 ^b^	18.98 ± 0.57 ^a^	**

Note: Values are expressed as mean ± SD based on three replicate tanks per treatment. T_1_ = 10 eels 250 L^−1^ of water; T_2_ = 15 eels 250 L^−1^ of water; and T_3_ = 20 eels 250 L^−1^ of water. In each row, means followed by different letters indicate significant differences among treatments. The absence of letters denotes no significant difference. * indicates significance at *p* ≤ 0.05; ** indicates significance at *p* ≤ 0.01; NS indicates non-significance (*p* > 0.05).

**Table 2:** Proximate composition (moisture, crude protein, crude lipid, and ash contents) of *A. marmorata* at the end of the experiment in an indoor media-based aquaponic system.

Component	T_1_	T_2_	T_3_	Significance
Moisture (%)	62.14 ± 0.51 ^c^	64.37 ± 0.85 ^b^	67.97 ± 0.69 ^a^	**
Crude protein (%)	16.77 ± 0.61 ^b^	17.53 ± 0.31 ^ab^	18.24 ± 0.18 ^a^	**
Crude lipid (%)	9.26 ± 0.15 ^a^	8.53 ± 0.36 ^a^	4.94 ± 0.74 ^b^	**
Ash (%)	1.93 ± 0.05 ^b^	2.42 ± 0.12 ^a^	2.30 ± 0.11 ^a^	**

Note: Values are expressed as mean ± SD, based on three replicate tanks per treatment. T_1_ = 10 eels 250 L^−1^ of water; T_2_ = 15 eels 250 L^−1^ of water; and T_3_ = 20 eels 250 L^−1^ of water. In each row, means followed by different letters indicate significant differences among treatments. ** indicates significance at *p* < 0.01.

**Table 3:** Growth performance of *B. rapa* var. *pekinensis* in an indoor media-based aquaponics system.

Traits	T_1_	T_2_	T_3_	Significance
Initial plant length (cm)	22.36 ± 0.68	22.93 ± 0.48	22.37 ± 0.70	NS
Final plant length (cm)	28.33 ± 1.68 ^a^	29.64 ± 1.67 ^a^	24.39 ± 2.32 ^b^	*
Initial shoot length (cm)	10.99 ± 0.31	11.42 ± 0.06	11.59 ± 0.57	NS
Final shoot length (cm)	12.85 ± 1.09	13.27 ± 2.58	11.78 ± 0.39	NS
Initial root length (cm)	11.43 ± 0.99	11.51 ± 0.51	10.78 ± 0.40	NS
Final root length (cm)	15.48 ± 1.18	16.36 ± 1.29	12.62 ± 1.99	NS
Leaf number	10.25 ± 4.10	10.65 ± 4.29	8.65 ± 2.27	NS
Chlorophyll (SPAD)	38.96 ± 9.65	42.18 ± 10.73	37.37 ± 9.00	NS

Note: Values are expressed as mean ± SD, based on three replicate tanks per treatment. T_1_ = 10 eels 250 L^−1^ of water; T_2_ = 15 eels 250 L^−1^ of water; and T_3_ = 20 eels 250 L^−1^ of water. In each row, means followed by different letters indicate significant differences among treatments. The absence of letters denotes no significant difference. * indicates significance at *p* < 0.05; NS indicates non-significance (*p* > 0.05).

## Data Availability

The data presented in this study are available upon request from the corresponding author.

## Abbreviations

The following abbreviations are used in this manuscript:

% LG	Percent length gain
% WG	Percent weight gain
AOAC	Association of Official Analytical Chemists
Ca	Calcium
CO_2_	Carbon dioxide
DMRT	Duncan’s Multiple Range Test
DO	Dissolved oxygen
DWG	Daily weight gain
EA	Eel ash content
EC	Electrical conductivity
ECL	Eel crude lipid
ECP	Eel crude protein
EM	Eel moisture content
EP	Eel production
FCR	Feed conversion ratio
FE	Feed efficiency
FI	Feed intake
FR	Feeding rate
h	Hour
HSI	Hepatosomatic index
K	Potassium
LG	Length gain
Mg	Magnesium
min	Minute
NH_3_	Ammonia
NH_4_	Ammonium
NO_2_	Nitrite
NO_3_	Nitrate
PER	Protein efficiency ratio
pH	Potential of hydrogen
PO_4_	Phosphate
S	Sulfur
SD	Standard deviation
SGR	Specific growth rate
SR	Survival rate
TAN	Total ammonia nitrogen
TDS	Total dissolved solids
WG	Weight gain
